# Supplementary material for: Alk1 acts in non-endothelial VE-cadherin+ perineurial cells to maintain nerve branching during hair homeostasis
Source: Nat Commun. 2023 Sep 12;14:5623. doi: 10.1038/s41467-023-40761-5 (PMC10497554; doi:10.1038/s41467-023-40761-5)
Supplement: Supplementary file 1 — Supplementary Information [file 41467_2023_40761_MOESM1_ESM.pdf]

Supplementary information

Alk1 acts in non-endothelial VE-cadherin$^+$ perineurial cells to maintain nerve branching during hair homeostasis

Gopal Chovatiya[1], Kefei Nina Li[1], Jonathan Li[1], Sangeeta Ghuwalewala[1] and Tudorita Tumbar[1,*]

[1]Department of Molecular Biology and Genetics, Cornell University, Ithaca, New York, USA.

[*]Correspondence: tt252@cornell.edu

Supplemental Figures

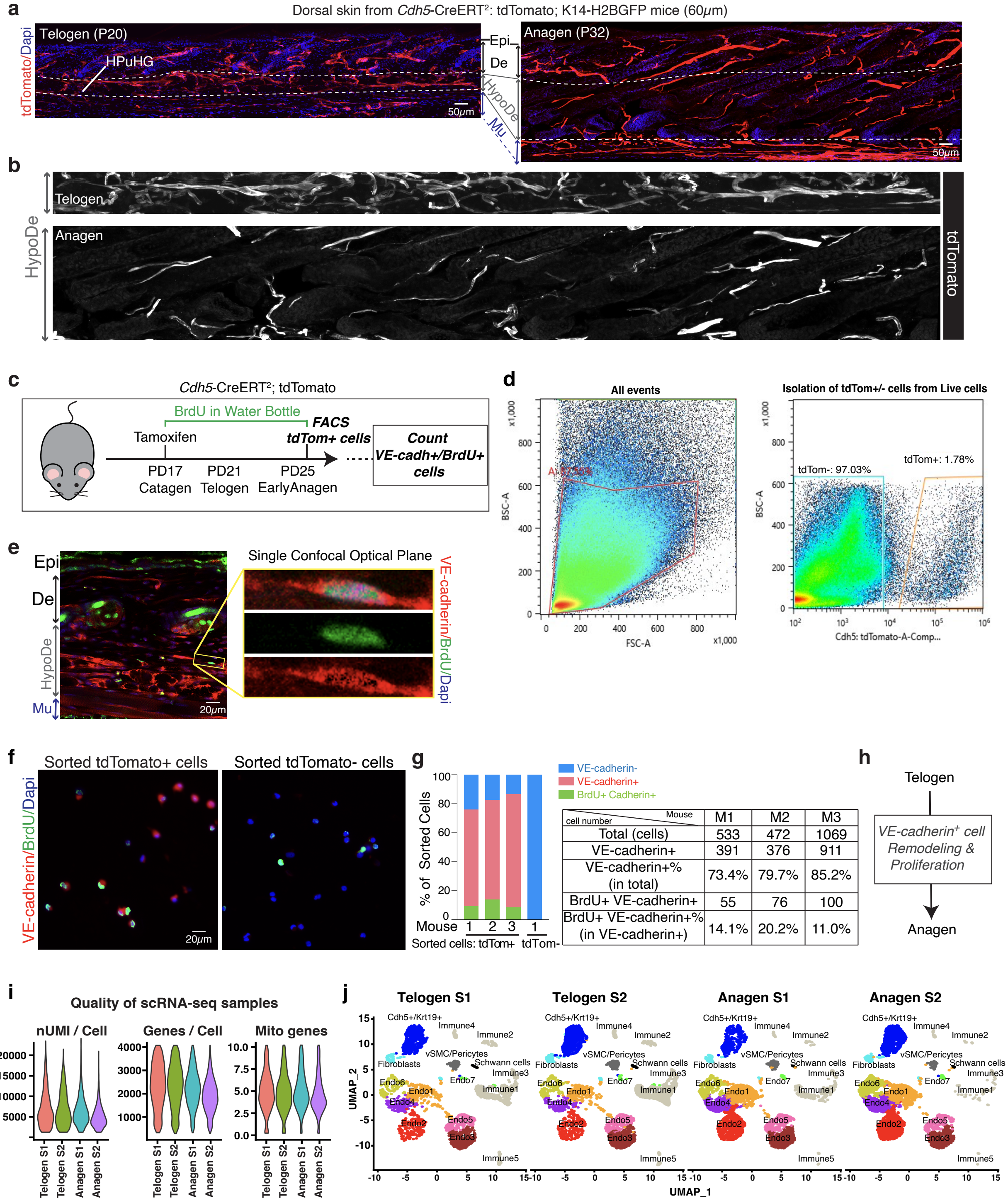

Supplementary Fig. 1, related to Fig. 1. Proliferation of VE-cadherin^{+} cells *in vivo* and their FACS isolation for scRNA-seq analysis.

a Maximal projections of confocal stack images showing 60μm dorsal skin sections at telogen and anagen from *Cdh5*-CreERT^{2}; tdTomato (red); *Krt14*-H2BGFP mice (green channel not shown).

HPuHG area is circled in a white dotted line. Scale bar 50µm. HypoDe, hypodermis; Mu, muscle; De, dermis, Epi, epidermis. (*n* = 3 biologically independent samples). **b** Single channel image (tdTomato) of cropped HPuHG region from (a). Note dispersion of HPuHG in the hypodermis area (HypoDe) area. **c** Schematic for tamoxifen induction and sample collection for BrdU-pulse experiment. **d** Gating strategy for isolation of $tdTomato^+$ cells from *Cdh5*-$CreERT^2$; tdTomato mice for BrdU incorporation assay *in vivo*. **e** Confocal single optical plane of 30µm thick skin sections from BrdU-pulsed mice stained for VE-cadherin (red), BrdU (green) and Dapi (blue). Enlarged cropped images on the right show BrdU in $VE\text{-}cadherin^+$ cell. (*n* = 3 biologically independent samples). **f** Sorted tdTomato-positive and tdTomato-negative cells stained with VE-cadherin (red), BrdU (green) and Dapi (blue). **g** Quantification for VE-cadherin and BrdU stainings co-localization in sorted cells from panel (f). n=3 mice. **h** Summary showing $tdTomato^+$ cells undergo remodeling and proliferation from telogen to anagen transition *in vivo*. **i** Quality of selected cells from two mouse replicates per stage that were used for the scRNA-seq analysis, nUMI/Cell - Unique Molecular Identifiers detected per cell, Genes/Cell - number of genes detected per cell, and Mito genes – a percentage of mitochondrial genes per cell, S – sample. **j** First-level UMAP clustering identified a total of 16 populations in both replicates S1 and S2 at each stage (the combined UMAP was split by sample). Source data are provided as a Source Data file.

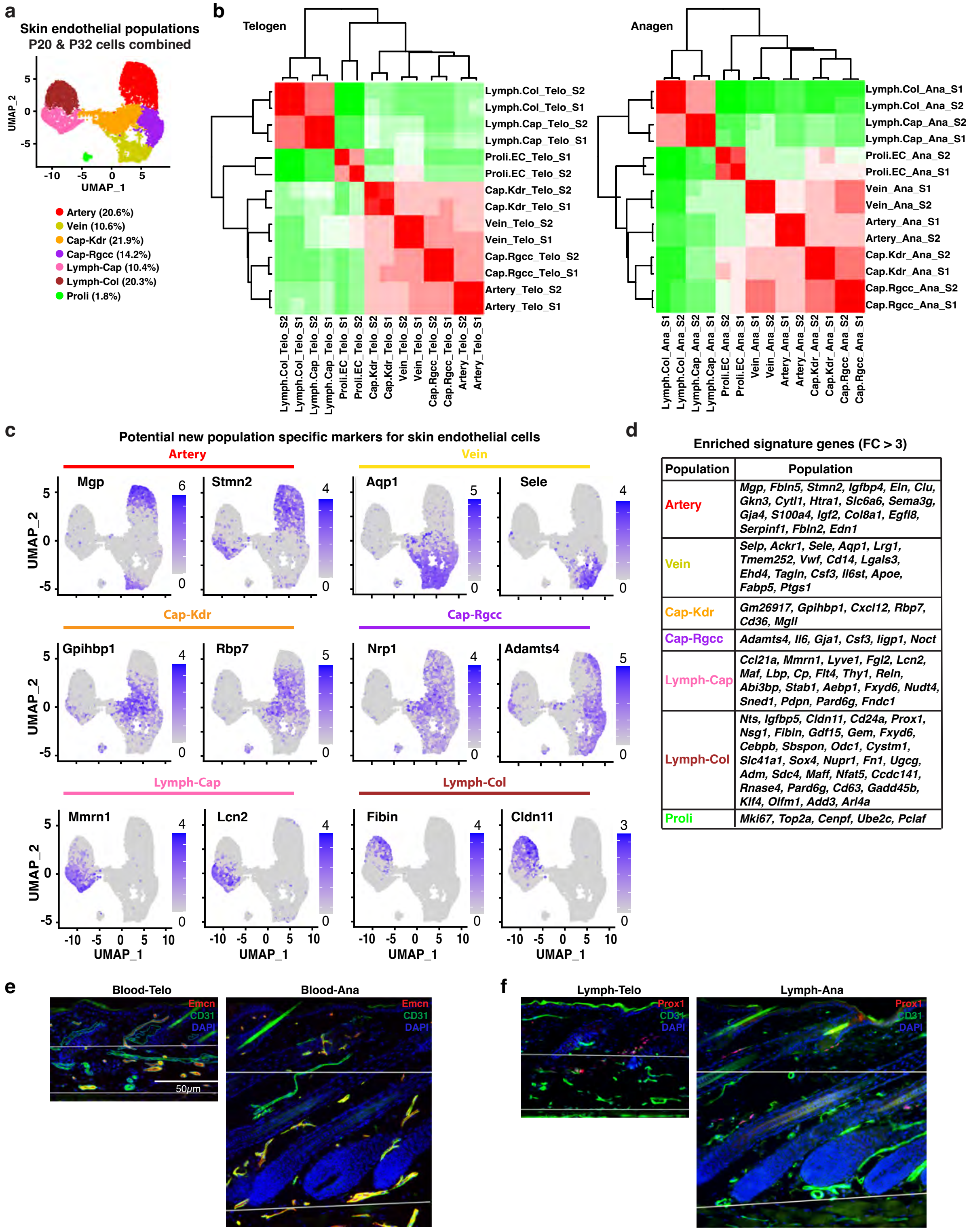

Enriched signature genes (FC > 3)

Population	Population
Artery	*Mgp, Fbln5, Stmn2, Igfbp4, Eln, Clu, Gkn3, Cytl1, Htra1, Slc6a6, Sema3g, Gja4, S100a4, Igf2, Col8a1, Egfl8, Serpinf1, Fbln2, Edn1*
Vein	*Selp, Ackr1, Sele, Aqp1, Lrg1, Tmem252, Vwf, Cd14, Lgals3, Ehd4, Tagln, Csf3, Il6st, Apoe, Fabp5, Ptgs1*
Cap-Kdr	*Gm26917, Gpihbp1, Cxcl12, Rbp7, Cd36, Mgll*
Cap-Rgcc	*Adamts4, Il6, Gja1, Csf3, Iigp1, Noct*
Lymph-Cap	*Ccl21a, Mmrn1, Lyve1, Fgl2, Lcn2, Maf, Lbp, Cp, Flt4, Thy1, Reln, Abi3bp, Stab1, Aebp1, Fxyd6, Nudt4, Sned1, Pdpn, Pard6g, Fndc1*
Lymph-Col	*Nts, Igfbp5, Cldn11, Cd24a, Prox1, Nsg1, Fibin, Gdf15, Gem, Fxyd6, Cebpb, Sbspon, Odc1, Cystm1, Slc41a1, Sox4, Nupr1, Fn1, Ugcg, Adm, Sdc4, Maff, Nfat5, Ccdc141, Rnase4, Pard6g, Cd63, Gadd45b, Klf4, Olfm1, Add3, Arl4a*
Proli	*Mki67, Top2a, Cenpf, Ube2c, Pclaf*

Supplementary Fig. 2, related to Fig. 1 and 2. Decoding identity of single cell populations and counting of blood and lymphatic ECs *in vivo*.

a UMAP plot showing 7 endothelial populations after computationally removing the non-ECs followed by re-clustering (all four samples combined before generating UMAP plot). **b** Comparison of gene expression matrix from two replicates for all 7 endothelial populations that

showed a high correlation between samples at each stage. **c** Feature plots showing expression and distribution of selected signature genes for each endothelial population. **d** Table for top upregulated signature genes for each population. (FC>3). **e, f** Immunofluorescence staining images of 8µm thin skin section from PD20 (telogen) and PD32 (anagen) mice stained with either Endomucin (EMCN, red), CD31 (green) and Dapi (blue) (e) or Prox1 (red), CD31 (green) and Dapi (blue) (f). The whole skin was divided into dermis and hypodermis by a white line and the stained structures were encircled for nuclei counting purpose, as represented in Blood-Telo image. Scale bar 50µm. (*n* = 3 biologically independent samples).

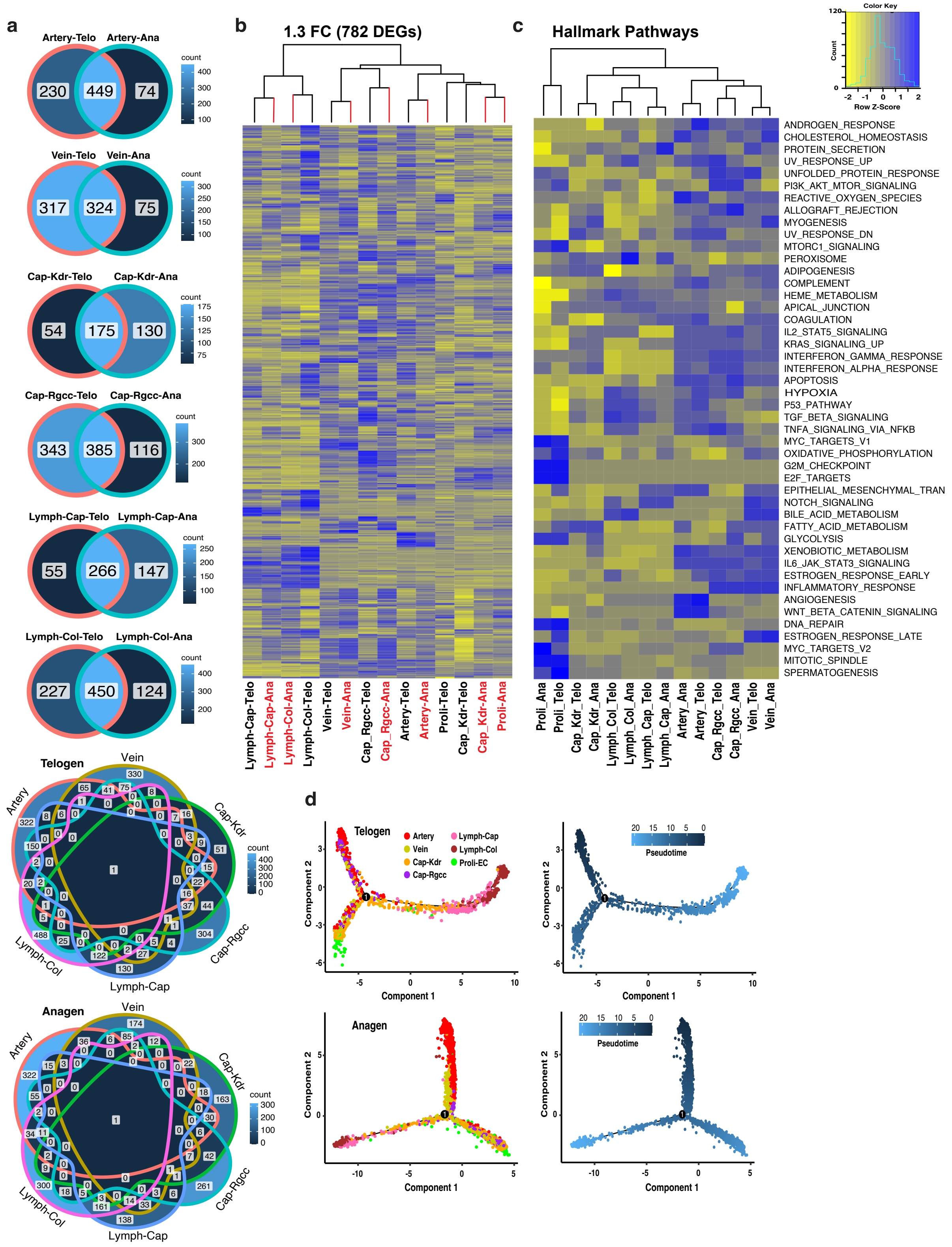

Supplementary Fig. 3, related to Fig. 2. Functional analysis on endothelial populations.

a Venn diagrams for signature genes (log2FC>0.25) at indicated stages. The signature genes were obtained by comparing each population with the rest of the populations at the same stage. The first

six plots show individual populations at two stages; last two plots show all populations at each stage. **b** Heatmap clustering of 782 DEGs (FC>1.3) showing population clustering and extent of gene expression changes between two stages (Raw values of DEGs in each population were extracted using *FetchData* function). **c** Raw hallmark pathway analysis using all detected genes at both stages. The enrichment score was compared using the heatmap2 function in R. **d** Single-cell transcriptome trajectory reconstruction of EC populations using Monocle2 to predict lineage transition path. Pseudotime coloring indicates ground state in dark blue, corresponding to artery. Source data are provided as a Source Data file.

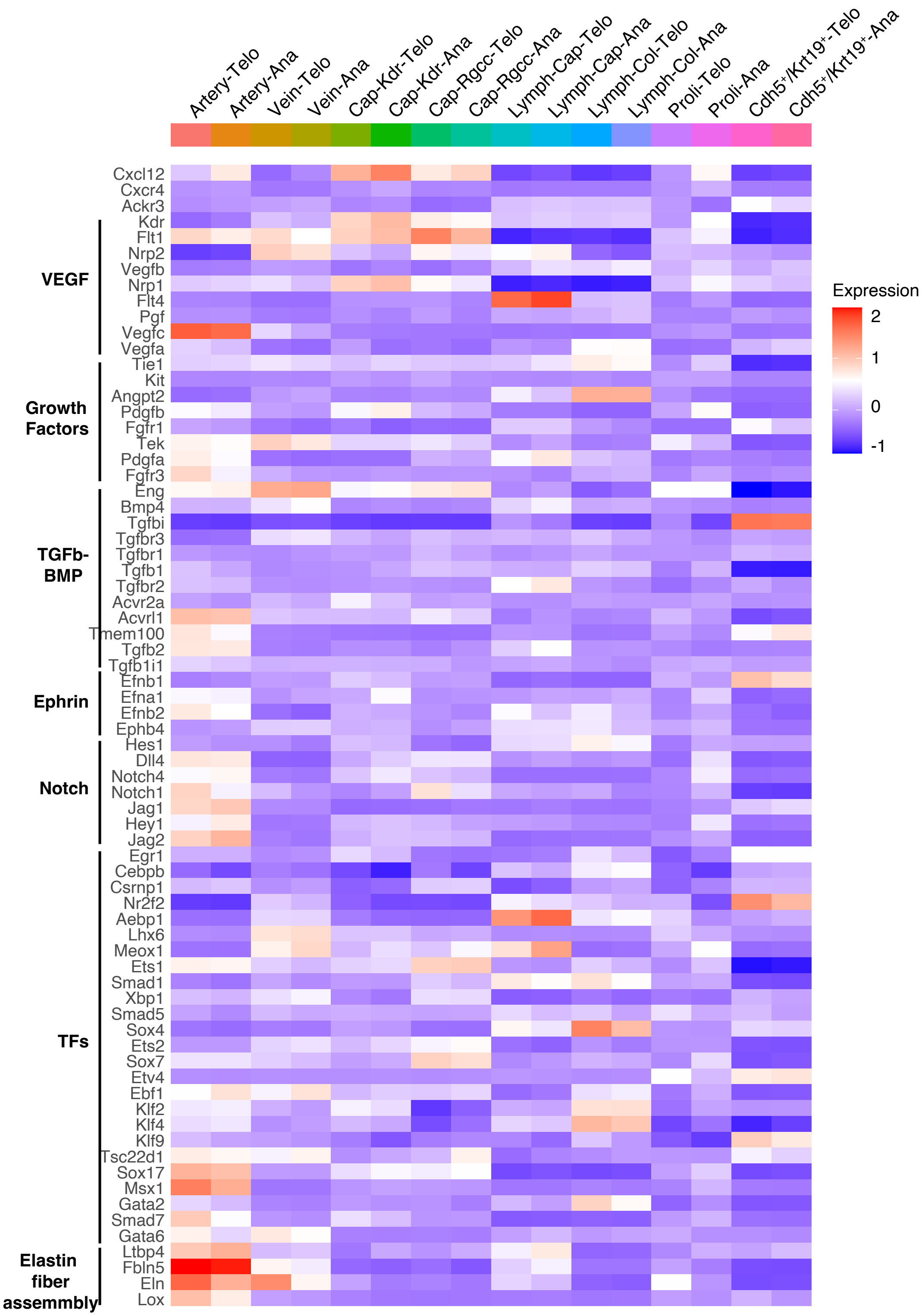

Supplementary Fig. 4, related to Fig. 2. Expression of angiogenesis-related factors in skin EC populations.

Heatmap showing expression of various gene classes known to be involved in angiogenesis. The gene list was extracted from Brulois *et al.*, 2020[1].

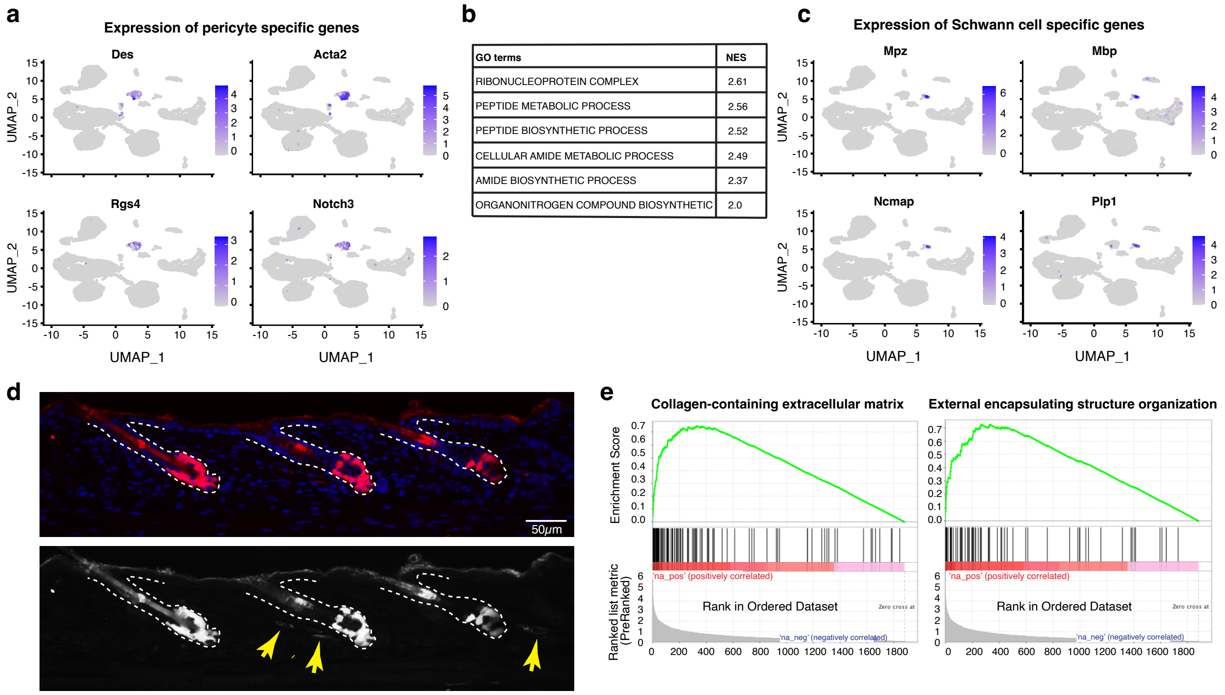

GO terms	NES
RIBONUCLEOPROTEIN COMPLEX	2.61
PEPTIDE METABOLIC PROCESS	2.56
PEPTIDE BIOSYNTHETIC PROCESS	2.52
CELLULAR AMIDE METABOLIC PROCESS	2.49
AMIDE BIOSYNTHETIC PROCESS	2.37
ORGANONITROGEN COMPOUND BIOSYNTHETIC	2.0

Supplementary Fig. 5, related to Fig. 3. Characterization of VE-cadherin expressing non-EC populations.

a UMAP plots showing selective expression of pericyte-specific genes *Des*, *Acta2*, *Rgs4* and *Notch3*. **b** Table showing Normalized Enrichment Score (NES) for top GO categories for pericytes enriched at anagen. DEGs at anagen (FC>1.3) was used for GO-term analysis. **c** UMAP plots for the schwann cell-specific genes *Mpz*, *Mbp*, *Ncmap* and *Plp1*. **d** Immunofluorescence images showing labeling efficiency in and outside hair follicles using *Krt19*-CreERT;tdTomato mice. ($n = 4$ biologically independent samples). **e** Gene Set Enrichment Analysis (GSEA) for *Cdh5*$^{+}$/*Krt19*$^{+}$ population using all detected genes highlighting enrichment for collagen-containing extracellular matrix, encapsulation, integrin binding and adhesion-associated functions indicative of a strong mesenchymal phenotype.

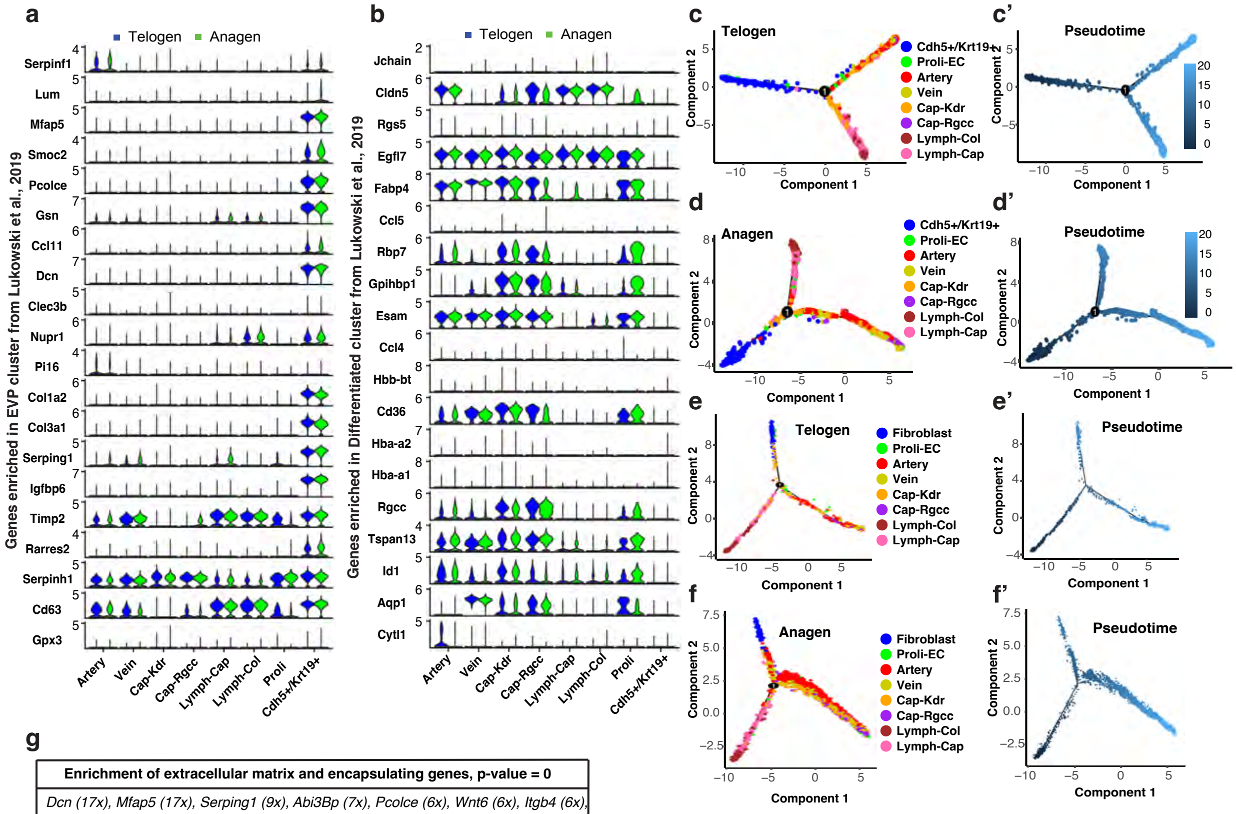

g

Enrichment of extracellular matrix and encapsulating genes, p-value = 0
Dcn (17x), Mfap5 (17x), Serping1 (9x), Abi3Bp (7x), Pcolce (6x), Wnt6 (6x), Itgb4 (6x), Sbspon (6x), Ogn (6x), Tgfbi (5x), Thbs4 (5x), Vtn (5x), Ntng1 (5x), Smoc2 (4x), Col5A3 (4x), Matn2 (4x), Adamts2 (4x), Col6A1 (4x), Omd (4x), Postn (4x), Sfrp1 (4x), Col6A3 (3x), Lgals1 (3x), Flrt2 (3x), Sdc2 (3x), Ecm2 (3x), Lamb2 (3x), Gpc3 (3x), Col3A1 (3x), Prelp (3x), Bgn (3x), Efemp1 (x), Thbs1 (3x), Col1A2 (3x), Adamtsl3 (3x), Mmp14 (3x), Nid1 (3x), Rarres2 (3x), Col5A2 (2x), Cspg4 (2x), Nid2 (2x), Tnxb (2x), Gpc6 (2x), F3 (2x), Timp2 (2x), Col6A2 (2x), Egfl6 (2x), Adamts5 (2x), Vit (2x), S100A6 (2x), Lum (2x), Col1A1(2x)

Supplementary Fig. 6, related to Fig. 4. scRNA-seq analysis testing the *Cdh5*$^{+}$*/Krt19*$^{+}$ population as a putative endovascular progenitor (EVP).

a Violin plots showing expression of top 20 endovascular progenitors (EVP) genes from Lukowski *et al*., 2019[2] in all 7 EC populations with *Cdh5*$^{+}$/*Krt19*$^{+}$ population. **b** Violin plots showing expression of top 20 differentiated (D) population's genes from Lukowski *et al*., 2019[2] in all 7 EC populations with *Cdh5*$^{+}$/*Krt19*$^{+}$ population. **c-d'** Single-cell trajectory reconstruction of EC populations with *Cdh5*$^{+}$/*Krt19*$^{+}$ population using Monocle placed *Cdh5*$^{+}$/*Krt19*$^{+}$ population at ground state at both the stages, but not the control fibroblast population, as shown in **e-f'**. **g** Table of enriched genes in *Cdh5*$^{+}$/*Krt19*$^{+}$ population that are known to be associated with ECM and encapsulation functions.

a

Details of FACS sorted cells used for qRT-PCR in Fig.7

Genotype	Sex	PD at tamoxifen injected	PD at mice was Sacrificed	# of tdT+ cells sorted	RNA quality (RIN)
CT-1	F	PD17	PD20	55K	7.7
CT-2	F	PD17	PD20	45K	8.4
CT-3	M	PD17	PD20	46K	7.4
Alk1KO-1	F	PD17	PD20	57K	7.6
Alk1KO-2	F	PD17	PD20	32K	8.3
Alk1KO-3	M	PD17	PD20	33K	9.4

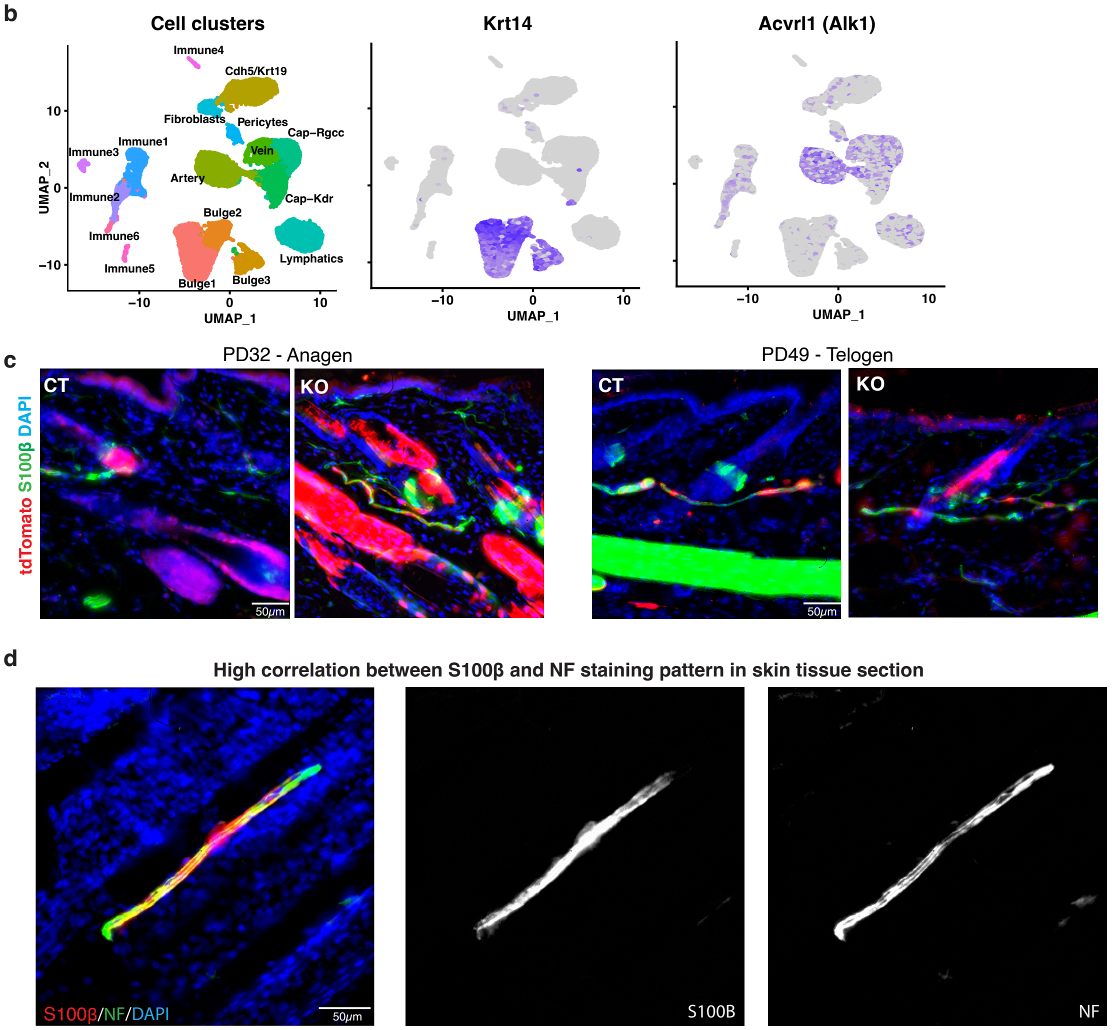

Supplementary Fig. 7, related to Fig. 7 and Fig. 8. Role of Alk1 in regulation of perineurial cell functions.

a Details of tdTomato$^+$ cell sorting from *Cdh5*-CreERT^2;tdTomato x Alk1$^{fl/fl}$ mice and RNA integrity values (RIN) of samples used for real-time PCR. **b** Data used to generate feature plots integrated the VE-cadherin (this study) and our HF bulge data from Chovatiya *et al*., 2021[3]. **c** Raw confocal Z-stack maximum projections illustrate expected anagen and telogen morphologies in mice from both CT (*Krt19*-CreERT;tdTomato) and KO (*Krt19*-CreERT;tdTomato x Alk1 fl/fl, TM induced at PD17) genotypes. Scale bar 50μm. ($n = 2$ biologically independent samples). **d**

Immunofluorescence staining showing high co-localization between S100β and NF in skin tissue sections. Scale bar 50µm. ($n = 3$ biologically independent samples).

Supplementary Table 1 - Classification of differentially expressed genes in Alk1 KO perineurial cells into different functional categories.

Alk1KO/CT (Δ>3x) in *Cdh5*⁺/*Krt19*⁺ cluster

Upregulated (20 genes)		Downregulated (33 genes)		
**Biosynthesis & Growth (11)**	**Others (9)**	**ECM associated (14)**	**Cytoskeleton & structural (7)**	**Protein process. & signalling (12)**
Ccn2	*Apod*	*Lgals1*	*Marcks*	*Lypd2*
Lars2	*Tsc22d3*	*Col1a1*	*Emp3*	*Nme2*
Cyp2d22	*Fmo2*	*Col3a1*	*Tmsb4x*	*Igfbp5*
Rps27	*Gm20594*	*Serpinh1*	*Krt19*	*Sec61b*
Rps27rt	*Pnpla2*	*Postn*	*Tuba1a*	*Maged2*
Igfbp4	*Tns1*	*Fstl1*	*S100a10*	*Ostc*
Hist1h1c	*Txnip*	*Pcolce*	*Rala*	*Ccl11*
Cyp27a1	*Xist*	*Col1a2*		*Pdia6*
Glul	*Trp53inp1*	*Vtn*		*Calm1*
Rpl9		*Abi3bp*		*Sbspon*
Rps28		*Ppib, Ppic*		*Anxa2*
		Fn1		*Serf2*
		Sparc		

References:

1. Brulois K, *et al.* A molecular map of murine lymph node blood vascular endothelium at single cell resolution. *Nature Communications* **11**, 3798 (2020).

2. Lukowski SW, *et al.* Single-Cell Transcriptional Profiling of Aortic Endothelium Identifies a Hierarchy from Endovascular Progenitors to Differentiated Cells. *Cell Rep* **27**, 2748-2758 e2743 (2019).

3. Chovatiya G, Ghuwalewala S, Walter LD, Cosgrove BD, Tumbar T. High-resolution single-cell transcriptomics reveals heterogeneity of self-renewing hair follicle stem cells. *Exp Dermatol* **30**, 457-471 (2021).
