# Supplementary material for: Alk1 acts in non-endothelial VE-cadherin+ perineurial cells to maintain nerve branching during hair homeostasis
Source: Nat Commun. 2023 Sep 12;14:5623. doi: 10.1038/s41467-023-40761-5 (PMC10497554; doi:10.1038/s41467-023-40761-5)
Supplement: Supplementary file 3 — Description of Additional Supplementary Files [file 41467_2023_40761_MOESM3_ESM.pdf]

Description of Additional Supplementary Data Files

Supplementary Data 1
First-level differentially expressed genes between all identified cell populations
Statistics is based on a non-parametric Wilcoxon rank sum test performed by the Seurat package

Supplementary Data 2
Differentially expressed genes between endothelial cell populations at telogen and anagen
Statistics is based on a non-parametric Wilcoxon rank sum test performed by the Seurat package

Supplementary Data 3
Differentially expressed genes between anagen and telogen for all endothelial cell populations

Supplementary Data 4
Enrichment scores for hallmark pathways for all endothelial cell populations at telogen and anagen

Supplementary Data 5
Differentially expressed genes for Schwann cell population between anagen and telogen
Statistics is based on a non-parametric Wilcoxon rank sum test performed by the Seurat package

Supplementary Data 6
Differentially expressed genes for $Cdh5^{+}$/$Krt19^{+}$ cell population between KO vs CT
Statistics is based on a non-parametric Wilcoxon rank sum test performed by the Seurat package

Supplementary Data 7
qPCR primers sequences for genes analyzed in Fig. 7h
