# Supplementary material for: Alk1 acts in non-endothelial VE-cadherin+ perineurial cells to maintain nerve branching during hair homeostasis
Source: Nat Commun. 2023 Sep 12;14:5623. doi: 10.1038/s41467-023-40761-5 (PMC10497554; doi:10.1038/s41467-023-40761-5)
Supplement: Supplementary file 11 — Reporting Summary [file 41467_2023_40761_MOESM11_ESM.pdf]

nature portfolio

Corresponding author(s): Tudorita Tumbar

Last updated by author(s): Jul 24, 2023

Reporting Summary

Nature Portfolio wishes to improve the reproducibility of the work that we publish. This form provides structure for consistency and transparency in reporting. For further information on Nature Portfolio policies, see our Editorial Policies and the Editorial Policy Checklist.

Statistics

For all statistical analyses, confirm that the following items are present in the figure legend, table legend, main text, or Methods section.

n/a	Confirmed	
☐	☒	The exact sample size (n) for each experimental group/condition, given as a discrete number and unit of measurement
☐	☒	A statement on whether measurements were taken from distinct samples or whether the same sample was measured repeatedly
☐	☒	The statistical test(s) used AND whether they are one- or two-sided<br>*Only common tests should be described solely by name; describe more complex techniques in the Methods section.*
☐	☒	A description of all covariates tested
☐	☒	A description of any assumptions or corrections, such as tests of normality and adjustment for multiple comparisons
☐	☒	A full description of the statistical parameters including central tendency (e.g. means) or other basic estimates (e.g. regression coefficient) AND variation (e.g. standard deviation) or associated estimates of uncertainty (e.g. confidence intervals)
☐	☒	For null hypothesis testing, the test statistic (e.g. F, t, r) with confidence intervals, effect sizes, degrees of freedom and P value noted<br>*Give P values as exact values whenever suitable.*
☒	☐	For Bayesian analysis, information on the choice of priors and Markov chain Monte Carlo settings
☒	☐	For hierarchical and complex designs, identification of the appropriate level for tests and full reporting of outcomes
☒	☐	Estimates of effect sizes (e.g. Cohen's d, Pearson's r), indicating how they were calculated

Our web collection on statistics for biologists contains articles on many of the points above.

Software and code

Policy information about availability of computer code

Data collection	Data collection methods were thoroughly described in the Methods section.<br>Briefly, following softwares were used for data collection in this study.<br>1) BD FACSDiva™ Software (BD Biosciences)<br>2) Cell Ranger (v6.0) - https://support.10xgenomics.com/single-cell-gene-expression/software/pipelines/6.0/installation<br>3) Microsoft office (2019)<br>4) LAS X (Leica Microsystems)<br>5) ZEN v3.0 (Carl Zeiss)

Data analysis	1) FlowJo™ Software, v10.5.0 (BD Biosciences)<br>2) Seurat (v4.0) - https://cloud.r-project.org/web/packages/Seurat/index.html<br>3) Harmony (v0.1.1) for single cell RNA-seq data integration<br>4) Monocle and Monocle 3 for trajectory analysis - https://cole-trapnell-lab.github.io/monocle3/<br>5) ImageJ2 (v2.3.0) for images stitching and analysis<br>6) GraphPad Prism (Prism 9) for making graphs<br>7) ggplots2 (v3.4.2) for visualization of Seurat analysis data<br>8) GSEA (v4.2.1) for enrichment analysis<br>9) Adobe Illustrator (2022) and Adobe Photoshop (2022) for figure preparation

For manuscripts utilizing custom algorithms or software that are central to the research but not yet described in published literature, software must be made available to editors and reviewers. We strongly encourage code deposition in a community repository (e.g. GitHub). See the Nature Portfolio guidelines for submitting code & software for further information.

Data

Policy information about availability of data

All manuscripts must include a data availability statement. This statement should provide the following information, where applicable:

- Accession codes, unique identifiers, or web links for publicly available datasets
- A description of any restrictions on data availability
- For clinical datasets or third party data, please ensure that the statement adheres to our policy

Raw data of scRNA-seq experiments have been deposited in the NCBI Gene Expression Omnibus (GEO) database under accession numbers GSE211381
Source codes describing single cell RNA-seq analysis is publicly available at https://github.com/GChovatiya/Chovatiya_et_al_Nat_Comm_SourceCode

Human research participants

Policy information about studies involving human research participants and Sex and Gender in Research.

Reporting on sex and gender	Not applicable
Population characteristics	Not applicable
Recruitment	Not applicable
Ethics oversight	Not applicable

Note that full information on the approval of the study protocol must also be provided in the manuscript.

Field-specific reporting

Please select the one below that is the best fit for your research. If you are not sure, read the appropriate sections before making your selection.

☒ Life sciences ☐ Behavioural & social sciences ☐ Ecological, evolutionary & environmental sciences

For a reference copy of the document with all sections, see nature.com/documents/nr-reporting-summary-flat.pdf

Life sciences study design

All studies must disclose on these points even when the disclosure is negative.

Sample size	For single cell RNA-sequencing experiments, we used 2 mice per stage/genotype. For other experiments, no predetermined sample size calculations was performed, but minimum 3 mice were used for each stage/condition. The sample size (n) of each experiment is provided in the corresponding figure legends.
Data exclusions	In single cell RNA-seq analysis, outliers cells that had genes expressed below 200 or above 5000, and had over 10% of the UMIs mapped to mitochondrial genes were removed.
Replication	All experiments (except scRNA-seq) were performed at least with three independent biological repeats. Information for each independent experiments is provided in the appropriate figure legends.
Randomization	No specific method was used. For in vivo studies, litter-mates controls were used whenever possible.
Blinding	Investigators were not blinded during data collection and analysis.

Reporting for specific materials, systems and methods

We require information from authors about some types of materials, experimental systems and methods used in many studies. Here, indicate whether each material, system or method listed is relevant to your study. If you are not sure if a list item applies to your research, read the appropriate section before selecting a response.

Materials & experimental systems

n/a	Involved in the study
☐	☒ Antibodies
☒	☐ Eukaryotic cell lines
☒	☐ Palaeontology and archaeology
☐	☒ Animals and other organisms
☒	☐ Clinical data
☒	☐ Dual use research of concern

Methods

n/a	Involved in the study
☒	☐ ChIP-seq
☐	☒ Flow cytometry
☒	☐ MRI-based neuroimaging

Antibodies

Antibodies used

Following primary antibodies were used in this study.
1) CD31 (1:100, BD Biosciences, 550274);
2) VE-cadherin (1:500, R&D Systems, AF1002);
3) LYVE1 (1:400, Thermo Scientific, 14-0443-82);
4) Prox1 (1:300, Abcam, ab199359);
5) BrdU (1:300, Abcam, ab6326);
6) Endomucin (1:300, Santa Cruz Biotechnology, sc-65495);
7) CD34 (1:100, BD Biosciences, 553731);
8) Laminin (1:500, Sigma-Aldrich, L9393)
8) S100B (1:400, Proteintech, 15146-1-AP);
9) Neurofilament Heavy chain (1:500, Millipore, AB5539).

Following secondary antibodies were used
1) Donkey Anti-Chicken IgY H&L (FITC) (1:500, abcam, ab63507)
2) Donkey Anti-Rabbit IgG H&L (FITC) (1:500, abcam, ab6798)
3) Donkey Anti-Rat IgG (H+L) (FITC) (1:500, Jackson ImmunoResearch Labs, 712-095-153)
4) Donkey Anti-Goat IgG H&L (Alexa Fluor® 488) (1:500, abcam, ab150129)
5) Donkey Anti-Goat IgG H&L (Alexa Fluor® 488) (1:500, abcam, ab150129)
6) Goat Anti-Rabbit IgG (H+L) (Alexa Fluor® 594 AffiniPure) (1:500, Jackson ImmunoResearch Labs, 111-585-003)
7) Goat Anti-Chicken IgY H&L (Alexa Fluor® 568) (1:500, abcam, ab175477)
8) Donkey Anti-Goat IgG H&L (Alexa Fluor® 568) preadsorbed (1:500, abcam, ab175704)

Validation

All antibodies were commercially sourced and we relied on the validation statements by manufacturers.
Appropriate titer for mouse skin tissue were determined independently for each antibody.

Animals and other research organisms

Policy information about studies involving animals; ARRIVE guidelines recommended for reporting animal research, and Sex and Gender in Research

Laboratory animals

Mice were housed under regular light/dark cycles (12 h light:12 h dark) at room temperature in animal facility at Cornell University.
We used following strains of Mice (mus musculus) in this study.
1) Rosa26-tdTomato mice (Jax strain # 007905), PD (postnatal day) 17-PD49
2) Cdh5-CreERT2 mice (MGI:3848982), PD17-PD49
3) K14-H2BGFP mice (MGI:5286142), PD17-PD32
4) Alk1flox/flox mice (MGI:4398901), PD17-PD49
5) Krt19-CreERT mice (Jax strain # 026925), PD17-PD49

Wild animals

No wild animals were used in this study.

Reporting on sex

Mouse sex was not considered in the study design.
Littermates were used as control whenever possible.

Field-collected samples

The study did not involve field samples.

Ethics oversight

All the mouse work was performed in accordance with the Cornell University Institutional Animal Care and Use Committee (IACUC)

guidelines (protocol no. 2007-0125).

Note that full information on the approval of the study protocol must also be provided in the manuscript.

Flow Cytometry

Plots

Confirm that:

- [x] The axis labels state the marker and fluorochrome used (e.g. CD4-FITC).
- [x] The axis scales are clearly visible. Include numbers along axes only for bottom left plot of group (a 'group' is an analysis of identical markers).
- [x] All plots are contour plots with outliers or pseudocolor plots.
- [x] A numerical value for number of cells or percentage (with statistics) is provided.

Methodology

Sample preparation	FACS isolation of VE-cadherin+ cells and its purity check was performed as previously described (Chovatiya et al., 2021). Briefly, tdTomato (Jax Stock #007905), Cdh5-CreERT2 and Krt14-H2BGFP mice were used for VE-cadherin+ cells and control keratinocytes isolation for single-cell RNA-seq or tdTomato+ cells from control (CT) and Alk1KO mice for qPCR validation. The VE-cadherin+ cells were labeled with tdTomato by injecting tamoxifen (200 μg/g) at postnatal day (PD)17, and dorsal skin was collected at PD20 or PD32. The dorsal skin was digested in collagenase and Dispase mixture as previously described. The dead cells were removed by LIVE/DEAD™ Fixable Aqua Dead Cell Stain Kit (ThermoFisher). FACS Aria (BD Biosciences) was used for the cell sorting. FACS data were analyzed with the FlowJo (FlowJo™ Software, v10.5.0, BD Biosciences).
Instrument	BD FACSAria Fusion
Software	BD FACSDiva™ Software
Cell population abundance	The Cdh5-CreERT2 labeled tdTomato+ population was in range of ~1-5% from total live cells.
Gating strategy	Unstained cells were used to set Voltage and size selection.<br>Single color controls were used to set gating for tdTomato+ cells.<br>Live cells were selected by LIVE/DEAD™ Fixable Aqua Dead Cell Stain (L34966, Invitrogen).

- [x] Tick this box to confirm that a figure exemplifying the gating strategy is provided in the Supplementary Information.
